# A High-Resolution LED Stimulator for Steady-State Visual Stimulation: Customizable, Affordable, and Open Source

**DOI:** 10.3390/s24020678

**Published:** 2024-01-21

**Authors:** Mónica Otero, Yunier Prieur-Coloma, Wael El-Deredy, Alejandro Weinstein

**Affiliations:** 1 Facultad de Ingeniería, Arquitectura y Diseño, Universidad San Sebastián, Santiago de Chile 8420000, Chile; monica.otero@uss.cl; 2 Centro BASAL Ciencia & Vida, Universidad San Sebastián, Santiago de Chile 8580000, Chile; 3 Brain Dynamics Laboratory, Universidad de Valparaíso, Valparaíso 2340000, Chile; yunier.prieur@gmail.com (Y.P.-C.); wael.el-deredy@uv.cl (W.E.-D.); 4 Escuela de Ingeniería Civil Biomédica, Facultad de Ingeniería, Universidad de Valparaíso, Valparaíso 2340000, Chile; 5 Advanced Center for Electrical and Electronic Engineering, Universidad Técnica Federico Santa María, Valparaíso 2340000, Chile

**Keywords:** steady-state visual stimulation, phase control, steady-state visual evoked potentials, customizable LED stimulator, PWM technique, open source LED stimulator

## Abstract

Visually evoked steady-state potentials (SSVEPs) are neural responses elicited by visual stimuli oscillating at specific frequencies. In this study, we introduce a novel LED stimulator system explicitly designed for steady-state visual stimulation, offering precise control over visual stimulus parameters, including frequency resolution, luminance, and the ability to control the phase at the end of the stimulation. The LED stimulator provides a personalized, modular, and affordable option for experimental setups. Based on the Teensy 3.2 board, the stimulator utilizes direct digital synthesis and pulse width modulation techniques to control the LEDs. We validated its performance through four experiments: the first two measured LED light intensities directly, while the last two assessed the stimulator’s impact on EEG recordings. The results demonstrate that the stimulator can deliver a stimulus suitable for generating SSVEPs with the desired frequency and phase resolution. As an open source resource, we provide comprehensive documentation, including all necessary codes and electrical diagrams, which facilitates the system’s replication and adaptation for specific experimental requirements, enhancing its potential for widespread use in the field of neuroscience setups.

## 1. Introduction

Sensory repetitive stimulation induces neural activity synchronization in populations of cortex neurons, which is reflected as distinct oscillatory patterns in the electroencephalogram (EEG) across various sensory modalities. In the case of visual stimulation, steady-state visually evoked potentials (SSVEPs) are neural responses elicited by visual stimuli oscillating at specific frequencies, providing valuable insights into the functional organization of visual processing and cognitive functions in health [1,2,3,4] and pathological conditions [5,6,7,8,9,10]. SSVEPs can be recorded from the scalp using EEG recordings, obtaining the maximum amplitude at the stimulation frequency, usually at occipital brain regions. The amplitude of the frequency components in SSVEP signals remains consistent over time, allowing for the reliable identification of stimulus frequency through frequency domain analysis [11]. The robust frequency characteristics of SSVEPs have led to the widespread utilization of the frequency tagging technique, which involves encoding multiple visual targets with distinct flickering frequencies [12,13]. This technique has found extensive applications in visual neuroscience and neural engineering domains.

In order to investigate SSVEPs, precise and customizable visual stimulation is essential. However, conventional displays have fixed sampling rates, limiting the number of frequencies that can be presented and potentially missing critical frequency components. The drawbacks mentioned above and the high cost of acquiring customizable light-emitting diode (LED) stimulators present significant challenges in experimental setups, limiting accessibility for numerous researchers.

Different types of visual stimuli can generate SSVEPs, including flickering checkerboard patterns, sinusoidal gratings, or even complex images modulated at specific frequencies [1]. Previous works have proposed visual stimulation paradigms using different modulation techniques [1]. Chang et al. proposed amplitude-modulated (AM) visual stimulation to reduce eye fatigue and the risk of epileptic seizures [14]. The visual stimuli were generated using two LED arrays, and the AM stimulus was designed to convey information at the alpha band within a high-frequency stimulus. In a study by Dreyer et al. [15], an LED driven by frequency-modulated (FM) signals was employed to elicit 10 Hz SSVEPs under various frequency modulation conditions, although the frequency resolution of the stimulation or the capability to control the phase of the stimulation were not tested. Other researchers have proposed frequency shift keying (FSK) and dual-frequency biased coding techniques to elicit the SSVEPs associated with flickering patterns at distinct frequencies [16,17]. However, these works focused on accurately decoding various commands in SSVEP-based BCI applications, and the stimuli were presented on a display screen, so the choice of frequencies was strictly limited by the screen´s refresh rate. Each type of stimulus has unique advantages and applications, enabling researchers to investigate various aspects of visual processing and cognitive functions. This article introduces a customizable and affordable LED stimulator to generate sinusoidal-like visual stimuli for SSVEPs. The LED stimulator proposed here was designed to provide a high degree of control over the parameters of the visual stimuli, including their luminance, frequency resolution, and wave phase, allowing researchers to tailor the stimulation to their specific experimental needs.

Controlling the phase of the visual stimulation is crucial for various experimental paradigms, particularly in electrophysiological studies. The ability to control the phase of the stimulation allows for the investigation of phase-dependent neural responses and their relationship to cognitive processes [18,19,20,21,22].

Pulse width modulation (PWM) is a modulation technique that generates variable-width pulses to encode the amplitude of an analog input signal [23]. Direct digital synthesis (DDS) is a technique to generate arbitrary analog signals using a digital circuit [24]. Combining the PWM and DDS techniques, the LED stimulator can produce visual stimulation signals resembling sinusoidal waves with precise, high-frequency resolution and end phase, with the advantage of requiring a simple LED driving circuit.

In this article, by leveraging the DDS and PWM techniques, we successfully produced visual stimuli at different frequencies while controlling the phase of both the initiation and termination of the sinusoidal-like signals emitted by the LEDs. Additionally, the LED stimulator described in this study enables synchronization with external EEG equipment, allowing the recording of triggers alongside neural activity. This feature is essential for accurate data analysis.

Furthermore, this article discusses the limitations of traditional stimulators, presenting a comprehensive exploration of the LED stimulator’s technical properties, including both hardware and software components. The advantages of employing a customizable LED stimulator are highlighted, emphasizing the significance of phase control and synchronization with external EEG recording equipment in advancing SSVEP research. Source codes and hardware schematics are available as open source LED stimulator designs for periodic stimulation. We also discussed the advantages of the LED stimulator proposed here in advancing our understanding of visual processing and contributing to developing new EEG-based strategies for assessing and diagnosing neurological disorders.

## 2. Materials and Methods

### 2.1. LED Stimulator

**Figure 1:**
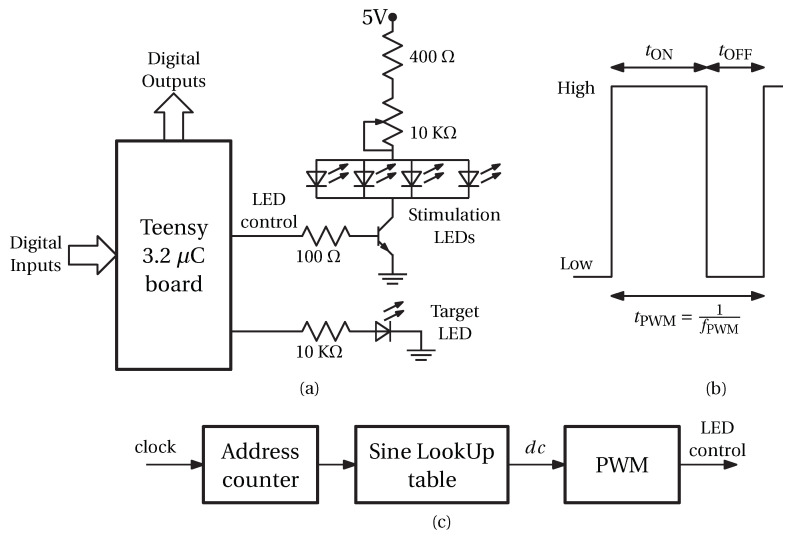
Block diagram of the stimulator. (**a**) The Teensy 3.2 µC board controls the stimulator. The LED control signal is a PWM digital output that turns the stimulation LEDs on and off through a transistor. The LEDs maximum intensities are adjusted using a potentiometer. A digital output controls the target LED. A set of general digital input and output signals is also available. (**b**) The PWM signal has two levels: low (LEDs off) and high (LEDs on). The duty cycle is defined as $dc=\frac{t_{\mathrm{ON}}}{t_{\mathrm{ON}}+t_{\mathrm{OFF}}}$. The PWM period is $t_{\mathrm{PWM}}=t_{\mathrm{ON}}+t_{\mathrm{OFF}}$, and its frequency is $f_{\mathrm{PWM}}=\frac{1}{t_{\mathrm{PWM}}}.$ (**c**) The LED control signal is generated by combining DDS and PWM techniques. A clock signal incrementally changes an address counter. This counter is used to index a lookup table that encodes duty cycles with the values of each sine sample. The duty cycles are transformed into the digital signal that controls the LEDs using the PWM microcontroller peripheral.

The LED stimulator was designed to facilitate the development of SSVEP experiments by presenting a sinusoidally varying light at a specific frequency customizable to each participant and terminating at different phases of the sinusoid. The proposed stimulator consists of two major components: a stimulator controller and an array of four stimulation LEDs, as shown in Figure 1a. The stimulator controller was designed using a Teensy 3.2 USB-based microcontroller development system (PJRC, Sherwood, OR, USA) that drives the stimulation LEDs using a PWM digital output and a transistor. In addition, a set of digital inputs and outputs is available to suit the needs of specific experiments. Table 1 shows the bill of materials (BOM) of the stimulator. The total cost is USD 64.3. All prices were obtained from the electronic part search engine Octopart [25].

The array of stimulation LEDs generates visual stimuli for the experiments. It consists of five white LEDs (part number YSL-R1042WC-D15 (China Yunsun LED Lighting Co., Shenzhen, China) with luminous intensity in the range of 13,000 to 15,000 mCd and a viewing angle in the range of 30 to 40 degrees. Four LEDs were positioned to form vertices of a 5 × 5 cm square at the center of a 50 × 50 cm black display. In our experimental setup, these four LEDs deliver the visual stimulus generating the SSVEP. The fifth LED of the array (target LED in Figure 1) was located at the center of the 5 × 5 cm square. The positioning of this fifth LED makes it possible to deliver transient stimuli simultaneously with steady-state stimulation while maintaining the area of the visual field covered by the stimuli generated only by the vertices LEDs. Since the fifth LED can also deliver continuous stimulation with equal or different temporal patterns than the vertices LEDs, spatial analyses of the SSVEP can be conducted when the fifth LED is used as a fixation point during experiments. Therefore, adding the fifth LED allows for meeting the specific requirements of different experimental designs. It is noteworthy that the LED stimulator is controlled such that different stimulation patterns can be arbitrarily directed simultaneously to one or a group of LEDs. Additionally, the maximum light intensity emitted by the LEDs can be adjusted using a potentiometer. The Teensy microcontroller and stimulation LEDs are powered by an external 5 V power supply.

It is worth noting that SSVEP can be elicited by the stimuli delivered only by one LED and that there is no standard regarding the number of LEDs used to generate SSVEP. In our system, to accommodate other LED arrangements or LED numbers, the power supply’s current capability and the transistor’s current capability need to be adjusted accordingly. The choice of the number of LEDs to be incorporated into a particular visual stimulator should match specific experimental requirements. For instance, the number of LEDs generating SSVEP can vary from one [15] to 30 [26]. Like the LED stimulator described here, other studies have conducted SSVEP experiments with four LED stimulation designs, where each LED delivers different stimulation frequencies [27,28].

We generated the four LED control signals by combining the DDS [24] with the PWM [23] techniques (Figure 1c). The LED control signal is a PWM signal with a high-low pattern, where a high level (LEDs are on) is followed by a low level (LEDs are off). In this pattern, the sum of the time the signal is high ($t_{\mathrm{ON}}$) and the time the signal is low ($t_{\mathrm{OFF}}$) is fixed, and it is called the PWM period, $t_{\mathrm{PWM}}$; its reciprocal is called the PWM frequency, $f_{\mathrm{PWM}}$, as shown in Figure 1b. For each PWM period, the duty cycle is defined as $\frac{t_{\mathrm{ON}}}{t_{\mathrm{ON}}+t_{\mathrm{OFF}}}=\frac{t_{\mathrm{ON}}}{t_{\mathrm{PWM}}}$. The duty cycle encodes the analog value to be generated. By having a high PWM frequency with respect to the visual system frequency response [29], this becomes a digital-to-analog conversion process. The duty cycle is generated using DDS, where the sinusoidal waveform with the desired frequency and end phase is stored in a lookup table. The lookup table values are read sequentially using an address counter incremented by one with each PWM period. The experiment stops when the address counter reaches the last value of the lookup table or it is reset if one wishes to deliver the stimulus again.

We implemented the generation of the LED control signal in the Teensy 3.2 board using the functions analogWriteFrequency and analogWrite and the class IntervalTimer  [30]. The functions analogWriteFrequency and analogWrite control the generation of the PWM signal. Class IntervalTimer creates a function that is called periodically. Listing 1 shows a code snippet with key implementation details (all codes are available at: https://doi.org/10.5281/zenodo.10223153, accessed on 1 December 2023). The first line includes a header file with an array containing the sine lookup table values. Line 2 creates an IntervalTimer dds object that is used in line 6 to attach function dds_update to a timer such that this function is executed every 1 ms. Line 5 uses analogWriteFrequency to set the PWM period $t_{\mathrm{PWM}}$ to 1 ms, or equivalently, to set the PWM frequency $f_{\mathrm{PWM}}$ to 1 kHz. Line 10 calls, from function dds, the function analogWrite. This function sets the PWM duty cycle to the value lookup_table[address_counter]. Variable ledPin defines the Teensy pin used to generate the LED control signal. Lines 11 to 13 increment address_counter and stop the experiment if the counter reaches the last address (constant MAX_ADDRESS) of the lookup table (the experimenter can change line 13 to handle what to do after presenting the stimulus according to the experiment details.
**Listing 1.** Code snippet to generate the LED control signal.
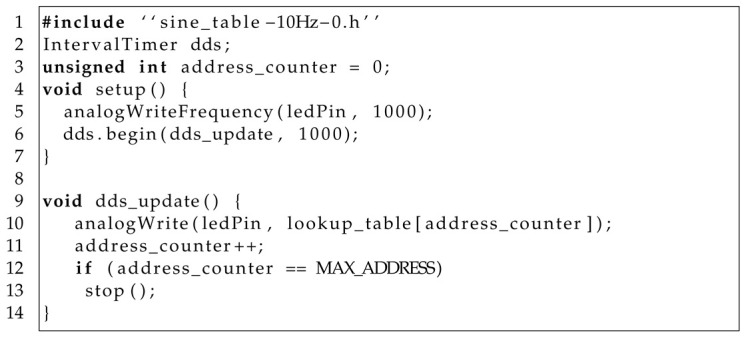



The function analogWrite sets the PWM with an integer value in the range of 0 to 256, where 0 corresponds to a duty cycle of 0 and 256 to a duty cycle of 1. For this reason, we fill the DDS lookup table with duty cycle (dc) values defined according to the following expression:

(1) $$dc=\frac{A\sin(\phi )+b}{256},$$

with A=100 and b=110 corresponding to duty cycles in the range of 0.04 to 0.82 to avoid too narrow pulses. Variable ϕ is set according to the desired frequency and end phase. We provide a Python script available at https://doi.org/10.5281/zenodo.10223153, (accessed on 1 December 2023) to create a header file with the lookup table corresponding to the desired stimulation parameters (frequency, end phase, and stimulation duration).

The software was developed in the Arduino programming environment. The freely available Arduino integrated development environment (IDE) provides functions and libraries for communicating and programming the Teensy boards. Thus, our code can be easily adapted to the specific requirements of each experiment. In particular, it is possible to extend the design to incorporate up to 12 independent stimulation patterns.

### 2.2. Experimental Design

We conducted four experiments to validate the frequency resolution and end phase of the stimulus generated with the LED stimulator. In the first two experiments, we measured the stimulus frequency resolution and end phase using a photodiode-based circuit. In the last two experiments, we validated the frequency resolution and end phase of the visual stimulus using EEG recordings of human participants who were exposed to the stimulus.

#### 2.2.1. Experiments 1 and 2

For experiment 1, we generated sinusoidal stimuli with frequencies from 9.5 to 10.4 Hz, with increments of 0.1 Hz and an end phase of stimulation of 0°. For experiment 2, we generated sinusoidal stimuli with a fixed frequency of 10 Hz and end phases of 0° to 315°, with increments of 45°.

**Figure 2:**
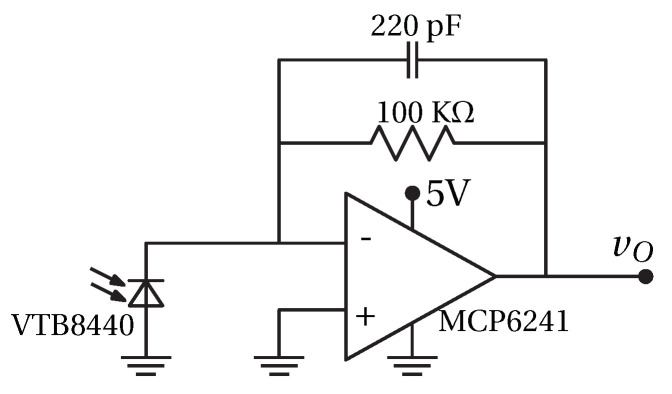
Light intensity measurement circuit. The LED light intensity is measured using a photodiode and a transimpedance amplifier. A logic analyzer measures the amplifier voltage.

For both experiments 1 and 2, we measured the stimulation LED light intensities with a transimpedance amplifier [31] (see Figure 2). The amplifier uses a VTB8440 (Excelitas Technologies, Waltham, MA, USA) visible light photodiode and an MCP6241 operational amplifier (Microchip Technology Incorporated, Waltham, AZ, USA). We adjusted the gain of the amplifier to get an output voltage of 5 V when the LEDs were on. We recorded the amplifier output voltage with Saleae Logic 8 (Saleae, San Francisco, CA, USA) with a sampling frequency of 25 MHz.

For experiment 1, we measured the stimulation frequency by performing a spectral analysis of the recorded LED light intensities. We estimated the recording’s power spectral density (PSD) using Welch’s method with a Hamming window [32] using the MATLAB 2021b implementation. The spectral analysis parameters were defined according to the desired physical frequency resolution, Δf, given by

(2) $$\Delta f=c\frac{f_{s}}{L},$$

where *c*, $f_{s}$, and *L* are the window-dependent constant (c=2 for a Hamming window), sampling rate, and window length, respectively [33]. We chose Δf=0.05 Hz and 5 segments with 50% overlap, leading to recordings with a total duration of 60 s.

For experiment 2, we measured the end phase, $\phi_{\mathrm{END}}$, of the stimulation by decoding the last two pulses of the LED light intensity measurements. First, we computed the duty cycle of these last two pulses as:

(3) $$dc_{N-1}=\frac{t_{N-1}}{t_{\mathrm{PWM}}}\;\mathrm{and}\;dc_{N}=\frac{t_{N}}{t_{\mathrm{PWM}}}.$$

In solving for ϕ in Equation (1), we get:

(4) $$\phi =\arcsin\left(\frac{256dc-b}{A}\right).$$

The sine function is not one-to-one; thus, the range of its inverse is limited to the [−90°, 90°] interval. Since we are interested in angles in the range of [0°, 360°], we used the sign of the duty cycle slope to compute the end phase:

(5) $$\phi_{\mathrm{END}}=\left\{\begin{array}{cc} \pi -\arcsin\left(\frac{256dc_{N}-b}{A}\right) & dc_{N-1}>dc_{N},\\ 2\pi +\arcsin\left(\frac{256dc_{N}-b}{A}\right) & dc_{N-1}\leq dc_{N}. \end{array}\right.$$

#### 2.2.2. Experiments 3 and 4

**Figure 3:**
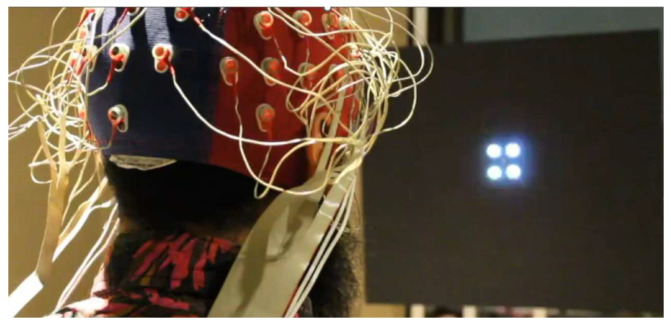
Image from the LED stimulator during an SSVEP experiment.

Experiments 3 and 4 were conducted to show the effect of visual stimulation generated by our LED stimulator on four participants (see Figure 3). Experiment 3 is related to the generation of sinusoidal-like stimulation at different frequencies, and experiment 4 is related to the variation in the phase at the stimulation offset.

The EEG was recorded from four healthy participants (age 25–26 years old, with two females and two males) following the experimental protocol described in [21]. The participants had either normal vision or vision corrected to normal and had no reported history of epilepsy, neurological, or psychiatric disorders. Before joining the study, all participants provided their written consent by signing a consent form. The experimental procedure was approved by the Research and Ethics Committee of the Universidad de Valparaíso (evaluation statement code CEC170-18) and adhered to the national regulations for human subject research and the principles outlined in the Declaration of Helsinki.

The participants were seated within a dimly illuminated, acoustically insulated, and electromagnetically shielded EEG chamber. Eyes-open resting-state EEG data were recorded for 5 min from 64 scalp locations using an Active II Biosemi acquisition system. The EEG signal was sampled at 8 kHz and processed offline using Brain Vision Analyzer 2.0 (Brain Products GmbH, Munich, Germany) according to the steps recommended in [34]. The EEG was acquired using the Biosemi default configuration for ground and reference electrodes, i.e., the Common Mode Sense (CMS) active electrode and the Driven Right Leg (DRL) passive electrode. During acquisition, a Cz reference was selected for visualization, although the EEG was actually acquired with respect to the CMS electrode.

The individual alpha frequency (IAF) [35,36] was the frequency selected for the visual stimulation and the generation of SSVEP. The IAF is the frequency with the highest power spectrum in the 8–14 Hz frequency band for each participant. For this purpose, the data were segmented into epochs of 5 s. The fast Fourier transform (FFT) was calculated for each segment using a frequency resolution of 0.125 Hz, and the mean power spectrum was computed [37].

For the SSVEP experiment, the participants were instructed to focus on the fifth LED located at the center of the LED stimulator, where the four white LEDs were located. The stimulator was placed at 70 cm from them. The area of the square of four LEDs subtended a visual angle of approximately 4° [21]. The vertical position of the LED stimulator was adjusted to align with the eye level of the participant.

During stimulation, the light intensity of the four LEDs followed a synchronized sinusoidal pattern of waxing and waning, matching the IAF of each participant [21]. We adjusted the maximum intensity of the stimulation according to the participant’s comfort, maintaining a suprathreshold minimum intensity.

Experiment 4 consisted of four experimental conditions in which the stimulus was set to finish at one of four possible phases of the sinusoid: 0°, 90°, 180°, and 270°, as in [21]. It is noteworthy that the stimulus onset did not vary among the experimental conditions and always started at phase zero, i.e., the mean intensity in the ascending ramp of the sinusoid. This was achieved by changing the stimulation time, with 500 ms being the maximum difference in the length between stimuli terminating at different phases. Evidence suggests that changes in the amplitude of the SSVEP cannot be systematically observed when stimulus differences are limited to 500 milliseconds. Studies of the adaptation of steady-state responses conducted in other sensory modalities [38,39] illustrate that while the steady-state response adapts to long-lasting continuous stimulation [38], adaptation is negligible when using intermittent, repetitive stimulation [39], as employed in the acquisition of the SSVEP (stimulus of 3–5 s in length, with an inter-stimulus interval of approximately the same duration).

The stimuli were presented in three blocks of 60 trials each. Within each block, the four experimental conditions were counterbalanced, and their order of presentation was randomized. The duration of the trials varied between 4.5 and 5 s, depending on the IAF of the participant and the specific experimental condition (phase of the stimulation at the offset). In order to avoid adaptation, the trials were followed by a resting interval of 2 s [34]. Each block lasted between 6.5 and 7 min, with 1 min of rest in between. The entire experimental session lasted approximately 45 min.

The EEG signals were processed similarly to the description provided above for resting-state EEG. The recordings were analyzed in the frequency domain, and the power of the SSVEP was defined as the mean power spectrum between trials.

## 3. Results

### 3.1. Experiments 1 and 2

**Figure 4:**
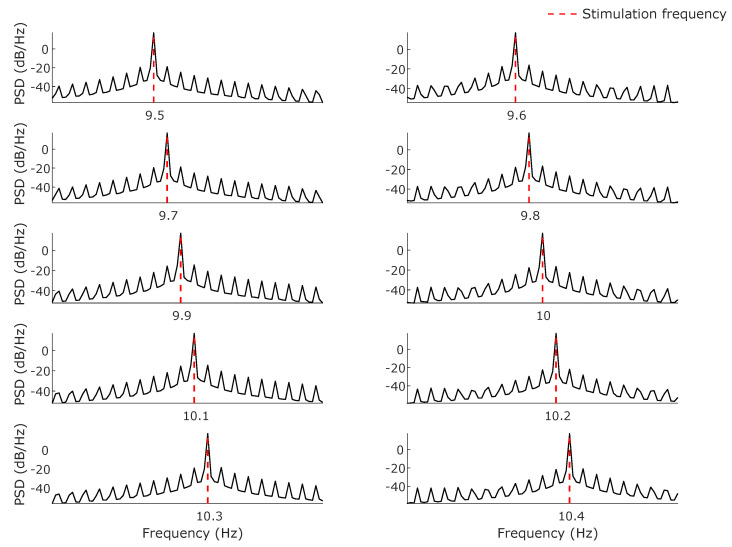
PSD estimation of the recorded LED light intensities during experiment 1. The PSDs were estimated using Welch’s method. The stimulation frequencies were set from 9.5 to 10.4 Hz with increments of 0.1 Hz.

**Figure 5:**
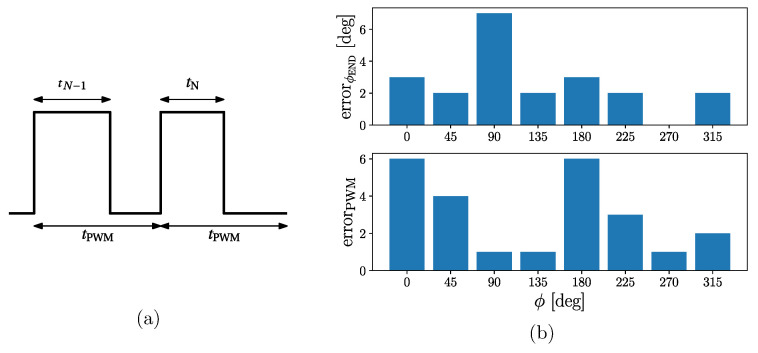
Phase error: (**a**) The phase is computed using the duty cycle of the last two PWM pulses, $dc_{N-1}=\frac{t_{N-1}}{t_{\mathrm{PWM}}}$ and $dc_{N}=\frac{t_{N}}{t_{\mathrm{PWM}}}$, respectively. (**b**) Measured PWM error (${\mathrm{error}}_{\mathrm{PWM}})$ and phase error (${\mathrm{error}}_{\phi }$) for reference phases 0°, 45°, …, 315°.

Figure 4 shows the estimated power spectral densities of the recorded signals during experiment 1. We can observe a clear peak in the PSD at the corresponding stimulation frequencies. Table 2 shows the results for experiment 2, including the nominal end phase $\phi_{\mathrm{END}}$ (for values 0°, 45°, …, 315°), the measured end phase $\hat{\phi_{\mathrm{END}}}$, and the absolute end phase error ${\mathrm{error}}_{\phi_{\mathrm{END}}}$, all in degrees. The phase is computed using the duty cycle of the last two PWM pulses (see Figure 5a). Table 2 also shows the corresponding PWM values (obtained through Equation (1)) for the nominal value PWM_END_, the measured value, $\hat{{\mathrm{PWM}}_{\mathrm{END}}}$, and the absolute error value, ${\mathrm{error}}_{\mathrm{PWM}}$. Figure 5b shows the end phase error value, ${\mathrm{error}}_{\phi_{\mathrm{END}}}$ (upper panel), and the PWM error value, ${\mathrm{error}}_{\mathrm{PWM}}$ (lower panel), for each end phase value. Note that there is a nonlinear relationship between ${\mathrm{error}}_{\phi_{\mathrm{END}}}$ and ${\mathrm{error}}_{\mathrm{PWM}}$, which is given by Equation (5).

Note that, as explained in Section 2.2.1, we measured the duty cycle and the corresponding PWM value using the photodiode-based circuit (Figure 2). The end phase was then computed using Equations (3) and (5). The PWM errors are small and were due to measurement noise. However, since the relationship between the PWM value and phase is nonlinear (see Equation (5)), a small error in the measurement of the PWM value may end in a relatively large error in the phase, as is the case for the nominal end phase of 90°.

### 3.2. Experiments 3 and 4

**Figure 6:**
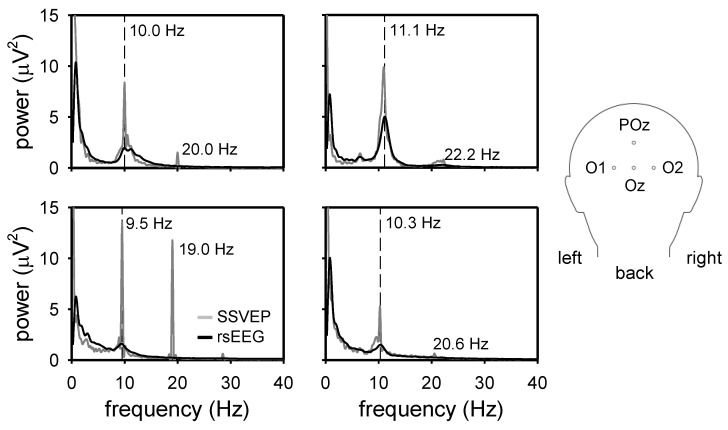
The mean of the power spectrum of four electrodes (O1, O2, POz, and OZ) from four participants exposed to visual stimulation generated with the LED stimulator. The inset at the right panel illustrates the position of the four electrodes in the conventional 10–20 EEG electrode placement scheme used in this study. The black and grey lines show the power spectrum of the resting-state EEG and the SSVEPs, respectively.

Our results showed that the stimulation generated the SSVEP at the expected frequency for all the study participants. Figure 6 shows the mean power spectral density in a pull of occipital electrodes (O1, O2, Oz, and POz) computed using EEG recordings from four participants at rest (black lines) and during the visual stimulation (grey lines), according to the methodology of Section 2.2.2.

We could effectively generate SSVEP at the participant’s IAF using the LED stimulator. In Figure 6 (black lines), we can observe the IAF, i.e., the peak in the range of 8–14 Hz for each participant. During stimulation, a visible increase in power at the IAF and its first harmonics can be observed (Figure 6 grey lines). This result is in accordance with the SSVEP literature [1,12,13].

Unlike the stimulus delivered by the LED array described in this study, the SSVEP is not a pure signal. Therefore, harmonics are expected when the SSVEP is analyzed in the frequency domain. The amplitude of these harmonics depends on factors such as the recording quality, noise spectral composition, and signal processing pipeline. In our study, a broad-band EEG spectrum is displayed to illustrate the SSVEP. Nevertheless, steady-state responses are typically detected in a narrow band around the fundamental frequency (F0), using statistical criteria to evaluate the signal-to-noise ratio (SNR). The amplitude of the steady-state response is defined as the EEG amplitude (or power) at the frequency the stimulus intensity varies, corresponding to the F0 of the steady-state response. The residual noise level (RNL) is computed by averaging the amplitude (or power) of a given number of FFT bins at each side of the F0. A threshold SNR is defined to determine if the steady-state response is elicited. Alternatively, the steady-state response and mean RNL are statistically compared using Hotelling’s T2 multivariate test, which considers both the amplitude and phase of the oscillation [38,39,40,41].

The results obtained from the EEG recordings using this LED stimulator to control the stimulus phase at the offset of stimulation can be found in [21]. In this work, the specific phase angles of the recorded EEG signal at this offset were computed. The study revealed that the EEG signal phases at the end of the stimulus were dispersed over a consistent range of values compared to the phase at the stimulus’s offset (see [21] for more details).

## 4. Discussion

In this article, we presented a high-resolution, affordable, and customizable LED stimulator for visual stimulation to generate SSVEPs. Our approach uses LED, DDS, and PWM techniques to generate a sinusoidal-like signal to control the light intensity and phase of the visual stimulation. The LED stimulator presented here is an open hardware and software device [42]; we provide the hardware schematics and all the stimulator software to promote the use of this approach and the replication of the study. The choice of Teensy as the stimulator board aligns with this goal, as it can be programmed in the Arduino environment [43,44] and is compatible with most operating systems [44]. The use of the Teensy microcontroller is also key for achieving this affordable solution. As can be seen from the BOM (see Table 1), the cost of our solution is under USD 100. In comparison, other similar solutions, such as the one presented by [27], use off-the-shelf equipment with costs exceeding USD 1000. This selection not only contributes to cost-effectiveness but also ensures excellent performance. Various initiatives for low-cost instrumentation have also opted for Teensy as their board controller of choice, as observed in [45,46,47].

### 4.1. Comparison with Other Available Systems

Other proposals for stimulators with visual stimulation purposes have been made using LED technology. In [48], a stimulus for the fly visual system was designed to present apparent motion stimuli. This stimulator technology was used to perform behavioral experiments with Drosophila. Furthermore, in the work of [15], a technique was developed using frequency-modulated (FM) visual stimuli for brain–computer interfaces, adapting the stimulation approach usually used in the auditory domain to evoke steady-state responses. In this approach, only 10 Hz was used as a modulated signal, and the SSVEPs recorded with EEG were compared to flicker stimulation in terms of perceptibility ratings. In [27], FM signals were proposed to elicit SSVEPS in a frequency range between 20 Hz and 29 Hz using independently flickering LEDs. However, this proposal does not explore the frequency resolution or its capability to control the phase of the stimulation. Furthermore, this approach involves the use of commercial devices with costs exceeding USD 1000 to control the LEDs, and the source codes and hardware schematics were not provided for replication.

In [49], an LED stimulator was created using an Arduino microcontroller for visual stimulation. This study demonstrated low latencies (1.2–2.4 ms) and established a linear relationship between the PWM duty cycle and the measured normalized light output, making this technique suitable for neuroscience experiments involving cue light tasks. The authors validated their system by assessing the light output quality and switching times. Compared to the system proposed here, a disadvantage of the approach in [49] is the computer requirements during experiment execution and the absence of triggers to mark the LED stimulation’s starting point. However, it is essential to note that this system was not designed for the sinusoidal modulation of LEDs, which is in contrast to the stimulator presented here.

The system proposed in [50] mainly focused on ensuring low power consumption for long-lasting battery applications. In the case of [50], unlike the sinusoidal-like stimulation proposed in this work, they used flickered signals at a rate of 20 Hz (with a fixed duty cycle of 50%). This approach does not allow for the control of the phase of the stimulation.

### 4.2. Applications in Advance Research in Visual Processing within the Context of Health and Disease

The novelty of the proposed LED stimulator lies in its capability to generate visual/flickering stimuli across a continuous frequency range, which is unrestricted by the discretized frequency range imposed by the refresh rate of conventional displays. This implies that the stimulation rate can be adjusted, coupled, or synchronized with the IAF.

Various flicker frequencies have been associated with diverse physiological responses and sensory outcomes [51] (see the review by [52]). SSVEP has demonstrated clinical relevance [12,53], with the effects on the EEG persisting beyond the stimulation period [21]. For instance, visual stimulation at the IAF has been identified as analgesic [54,55,56]. Rhythmic visual stimulation at an individual theta frequency has been shown to enhance memory formation [57], and the IAF has been correlated with pain sensitivity and tolerance [58,59]. Conversely, the authors of [60] found that a 10 Hz flicker interacting with the individual alpha peak interfered with attentional task performance, depending on the deviation of 10 Hz from the IAF.

Previous studies have reported slower IAFs in schizophrenia patients [61,62] and decreased SSVEP across frequencies and electrode locations in schizophrenia patients compared to healthy controls [5]. Moreover, variations in the IAF have been found among healthy controls, individuals with mild cognitive impairment, and Alzheimer’s disease (AD) patients [63,64,65]. By using visual stimulation at 15 Hz, the authors of [66] identified altered SSVEP in patients on the AD spectrum, with SSVEP amplitude predicting cognitive performance (i.e., Mini-Mental State Examination). Patients with SSVEP responses resembling those of control participants exhibited better cognitive performance.

The flexibility to drive brain oscillations at individualized frequencies provides enhanced control for studies in selective attention aging, neurodegeneration, and neuropsychiatry, where variability and alterations in EEG oscillations have been reported.

### 4.3. Applications within and beyond Visual Stimulation

Another important aspect of the proposed system is its versatility in terms of applications. This system is ready to generate sinusoidal-like visual stimuli for the generation of SSVEPs. The current system also provides an additional LED at the center of the square composed of the four driver LEDs, which could present transient visual stimulation at any time during the periodic stimulation. It is also possible to adapt the system to work with tactile stimulation delivered by solenoid stimulators, such as the Dancer Design tactor [67], with a minor change in the driver circuit, to generate steady-state somatosensory-evoked potentials [68,69].

## 5. Conclusions

This article presents an open source LED stimulator with a simple and affordable controlling system. Our system allows for the definition of arbitrary stimulation frequencies with a 0.1 Hz resolution and offers control of the stimulus phase. Moreover, the LED stimulator is also prepared to send triggers at the beginning (onset) and the end (offset) of the stimulation, ensuring synchronization between the recording system and the stimulation. Given that the software and circuits are accessible to the entire research community, the system can be personalized and configured to meet the diverse needs of different research hypotheses, where individualized frequencies of stimulation can be used, and the phase of the stimulation can be controlled at any time of the stimulation, making it a versatile solution for various scientific projects.

## Figures and Tables

**Table 1:** BOM for the stimulator.

Part	Quantity	Total Cost USD$
Tensy 3.2 board	1	23.2
Power Supply	1	9.0
Resistors	3	0.3
Potentiometer	3	3.8
LED	5	8.0
Transistor	1	0.3
Assorted components	1	20.0
	Total:	64.3

**Table 2:** Measured end phases and corresponding PWM values. The PWM values were measured directly using the photodiode circuit (Figure 2). The phase values were computed using Equations (3) and (5).

$\phi_{\mathrm{END}}$ [deg]	$\hat{\phi_{\mathrm{END}}}$ [deg]	${\mathrm{error}}_{\phi_{\mathrm{END}}}$ [deg]	PWM_END_	$\hat{{\mathrm{PWM}}_{\mathrm{END}}}$	${\mathrm{error}}_{\mathrm{PWM}}$
0°	357°	3°	110	104	6
45°	43°	2°	181	177	4
90°	83°	7°	210	209	1
135°	133°	2°	181	182	1
180°	177°	3°	110	116	6
225°	223°	2°	39	42	3
270°	270°	0°	10	9	1
315°	313°	2°	39	37	2

## Data Availability

https://doi.org/10.5281/zenodo.10223153 (accessed on 1 December 2023).

## Abbreviations

The following abbreviations are used in this manuscript:

LED	Light-emitting diode
EEG	Electroencephalogram
SSVEP	Steady-state visually evoked potential
PWM	Pulse width modulation
DDS	Direct digital synthesis
PSD	Power spectral density
IDE	Integrated development environment
IAF	Individual alpha frequency
AD	Alzheimer’s disease
FFT	Fast Fourier transform
SNR	Signal-to-noise ratio
RNL	Residual noise level
F0	Fundamental frequency

## References

[1] Norcia A.M., Appelbaum L.G., Ales J.M., Cottereau B.R., Rossion B. (2015). The steady-state visual evoked potential in vision research: A review. J. Vis..

[2] Kritzman L., Eidelman-Rothman M., Keil A., Freche D., Sheppes G., Levit-Binnun N. (2022). Steady-state visual evoked potentials differentiate between internally and externally directed attention. NeuroImage.

[3] Yang H., Paller K.A., van Vugt M. (2022). The steady state visual evoked potential (SSVEP) tracks “sticky” thinking, but not more general mind-wandering. Front. Hum. Neurosci..

[4] Zhang S., Gao X. (2019). The effect of visual stimuli noise and fatigue on steady-state visual evoked potentials. J. Neural Eng..

[5] Schielke A., Krekelberg B. (2022). Steady state visual evoked potentials in schizophrenia: A review. Front. Neurosci..

[6] Tabanfar Z., Firoozabadi M., Shankayi Z., Sharifi G. (2022). Screening of Brain Tumors Using Functional Connectivity Patterns of Steady-State Visually Evoked Potentials. Brain Connect..

[7] Danjou P., Viardot G., Maurice D., Garcés P., Wams E., Phillips K., Bertaina-Anglade V., McCarthy A., Pemberton D. (2019). Electrophysiological assessment methodology of sensory processing dysfunction in schizophrenia and dementia of the Alzheimer type. Neurosci. Biobehav. Rev..

[8] Murty D.V., Manikandan K., Kumar W.S., Ramesh R.G., Purokayastha S., Nagendra B., Ml A., Balakrishnan A., Javali M., Rao N.P. (2021). Stimulus-induced gamma rhythms are weaker in human elderly with mild cognitive impairment and Alzheimer’s disease. Elife.

[9] Lalancette E., Charlebois-Poirier A.R., Agbogba K., Knoth I.S., Jones E.J., Mason L., Perreault S., Lippé S. (2022). Steady-state visual evoked potentials in children with neurofibromatosis type 1: Associations with behavioral rating scales and impact of psychostimulant medication. J. Neurodev. Disord..

[10] Richard N., Nikolic M., Mortensen E.L., Osler M., Lauritzen M., Benedek K. (2020). Steady-state visual evoked potential temporal dynamics reveal correlates of cognitive decline. Clin. Neurophysiol..

[11] Nakanishi M., Wang Y., Wang Y.T., Mitsukura Y., Jung T.P. (2014). Generating visual flickers for eliciting robust steady-state visual evoked potentials at flexible frequencies using monitor refresh rate. PLoS ONE.

[12] Vialatte F.B., Maurice M., Dauwels J., Cichocki A. (2010). Steady-state visually evoked potentials: Focus on essential paradigms and future perspectives. Prog. Neurobiol..

[13] Herrmann C.S. (2001). Human EEG responses to 1–100 Hz flicker: Resonance phenomena in visual cortex and their potential correlation to cognitive phenomena. Exp. Brain Res..

[14] Chang M.H., Baek H.J., Lee S.M., Park K.S. (2014). An amplitude-modulated visual stimulation for reducing eye fatigue in SSVEP-based brain–computer interfaces. Clin. Neurophysiol..

[15] Dreyer A.M., Herrmann C.S. (2015). Frequency-modulated steady-state visual evoked potentials: A new stimulation method for brain–computer interfaces. J. Neurosci. Methods.

[16] Kimura Y., Tanaka T., Higashi H., Morikawa N. (2013). SSVEP-Based Brain–Computer Interfaces Using FSK-Modulated Visual Stimuli. IEEE Trans. Biomed. Eng..

[17] Ge S., Jiang Y., Zhang M., Wang R., Iramina K., Lin P., Leng Y., Wang H., Zheng W. (2021). SSVEP-Based Brain-Computer Interface With a Limited Number of Frequencies Based on Dual-Frequency Biased Coding. IEEE Trans. Neural Syst. Rehabil. Eng..

[18] Klimesch W., Sauseng P., Hanslmayr S. (2007). EEG alpha oscillations: The inhibition–timing hypothesis. Brain Res. Rev..

[19] Mathewson K.E., Lleras A., Beck D.M., Fabiani M., Ro T., Gratton G. (2011). Pulsed out of awareness: EEG alpha oscillations represent a pulsed-inhibition of ongoing cortical processing. Front. Psychol..

[20] Ronconi L., Melcher D. (2017). The role of oscillatory phase in determining the temporal organization of perception: Evidence from sensory entrainment. J. Neurosci..

[21] Otero M., Prado-Gutiérrez P., Weinstein A., Escobar M.J., El-Deredy W. (2020). Persistence of eeg alpha entrainment depends on stimulus phase at offset. Front. Hum. Neurosci..

[22] Fiene M., Schwab B.C., Misselhorn J., Herrmann C.S., Schneider T.R., Engel A.K. (2020). Phase-specific manipulation of rhythmic brain activity by transcranial alternating current stimulation. Brain Stimul..

[23] Mohan N., Undeland T.M., Robbins W.P. (2003). Power Electronics: Converters, Applications, and Design.

[24] Cordesses L. (2004). Direct digital synthesis: A tool for periodic wave generation (part 1). IEEE Signal Process. Mag..

[25] https://octopart.com/.

[26] Hwang H.J., Lim J.H., Jung Y.J., Choi H., Lee S.W., Im C.H. (2012). Development of an SSVEP-based BCI spelling system adopting a QWERTY-style LED keyboard. J. Neurosci. Methods.

[27] Dreyer A.M., Herrmann C.S., Rieger J.W. (2017). Tradeoff between user experience and BCI classification accuracy with frequency modulated steady-state visual evoked potentials. Front. Hum. Neurosci..

[28] Mouli S., Palaniappan R. (2020). DIY hybrid SSVEP-P300 LED stimuli for BCI platform using EMOTIV EEG headset. HardwareX.

[29] Plöchl M., Fiebelkorn I., Kastner S., Obleser J. (2022). Attentional sampling of visual and auditory objects is captured by theta-modulated neural activity. Eur. J. Neurosci..

[30] Lambert T.R. (2017). An Introduction to Microcontrollers and Embedded Systems. https://www.researchgate.net/profile/Tyler-Lambert/publication/340062601_Introduction_to_Microcontrollers_and_Embedded_Systems/links/5e7a4332299bf1f3873f8a24/Introduction-to-Microcontrollers-and-Embedded-Systems.pdf.

[31] Graeme J. (1995). Photodiode Amplifiers: Op Amp Solutions.

[32] Stoica P., Moses R.L. (2005). Spectral Analysis of Signals.

[33] Orfanidis S.J. (1995). Introduction to Signal Processing.

[34] Prado-Gutiérrez P., Otero M., Martínez-Montes E., Weinstein A., Escobar M.J., El-Deredy W., Zañartu M. (2019). A method for tracking the time evolution of steady-state evoked potentials. J. Vis. Exp..

[35] Tarasi L., Romei V. (2023). Individual alpha frequency contributes to the precision of human visual processing. J. Cogn. Neurosci..

[36] Klimesch W., Sauseng P., Gerloff C. (2003). Enhancing cognitive performance with repetitive transcranial magnetic stimulation at human individual alpha frequency. Eur. J. Neurosci..

[37] Manolakis D.G., Ingle V.K. (2011). Applied Digital Signal Processing: Theory and Practice.

[38] Prado-Gutierrez P., Castro-Fariñas A., Morgado-Rodriguez L., Velarde-Reyes E., Martínez A.D., Martínez-Montes E. (2015). Habituation of auditory steady state responses evoked by amplitude-modulated acoustic signals in rats. Audiol. Res..

[39] Prado-Gutierrez P., Martínez-Montes E., Weinstein A., Zañartu M. (2019). Estimation of auditory steady-state responses based on the averaging of independent EEG epochs. PLoS ONE.

[40] Valdes J.L., Perez-Abalo M.C., Martin V., Savio G., Sierra C., Rodriguez E., Lins O. (1997). Comparison of statistical indicators for the automatic detection of 80 Hz auditory steady state responses. Ear Hear..

[41] Savio G., Cardenas J., Pérez Abalo M., Gonzalez A., Valdes J. (2001). The low and high frequency auditory steady state responses mature at different rates. Audiol. Neurotol..

[42] Oellermann M., Jolles J.W., Ortiz D., Seabra R., Wenzel T., Wilson H., Tanner R.L. (2022). Open hardware in science: The benefits of open electronics. Integr. Comp. Biol..

[43] Zlatanov N. (2016). Arduino and open source computer hardware and software. J. Water Sanit. Hyg. Dev.

[44] Sebar L.E., Angelini E., Grassini S., Iannucci L., Parvis M. An op amp-less Electrochemical Impedance Spectroscopy System. Proceedings of the 2020 IEEE International Instrumentation and Measurement Technology Conference (I2MTC).

[45] Sebar L.E., Iannucci L., Angelini E., Grassini S., Parvis M. (2020). Electrochemical impedance spectroscopy system based on a teensy board. IEEE Trans. Instrum. Meas..

[46] Guidorzi P., Garai M. (2022). A Low-Cost System for Quick Measurements on Noise Barriers in Situ. IEEE Trans. Instrum. Meas..

[47] Lombardo L. (2023). Multi-platform solution for data acquisition. Acta IMEKO.

[48] Reiser M.B., Dickinson M.H. (2008). A modular display system for insect behavioral neuroscience. J. Neurosci. Methods.

[49] Teikari P., Najjar R.P., Malkki H., Knoblauch K., Dumortier D., Gronfier C., Cooper H.M. (2012). An inexpensive Arduino-based LED stimulator system for vision research. J. Neurosci. Methods.

[50] da Silva Pinto M.A., de Souza J.K.S., Baron J., Tierra-Criollo C.J. (2011). A low-cost, portable, micro-controlled device for multi-channel LED visual stimulation. J. Neurosci. Methods.

[51] Lea-Carnall C.A., Montemurro M.A., Trujillo-Barreto N.J., Parkes L.M., El-Deredy W. (2016). Cortical resonance frequencies emerge from network size and connectivity. PLoS Comput. Biol..

[52] Collura T.F., David Siever C. (2009). Auditory-Vsual Entrainment in Relation to Mental Health and EEG.

[53] Sabel B.A., Thut G., Haueisen J., Henrich-Noack P., Herrmann C.S., Hunold A., Kammer T., Matteo B., Sergeeva E.G., Waleszczyk W. (2020). Vision modulation, plasticity and restoration using non-invasive brain stimulation–An IFCN-sponsored review. Clin. Neurophysiol..

[54] Lopez-Diaz K., Henshaw J., Casson A.J., Brown C.A., Taylor J.R., Trujillo-Barreto N.J., Arendsen L.J., Jones A.K., Sivan M. (2021). Alpha entrainment drives pain relief using visual stimulation in a sample of chronic pain patients: A proof-of-concept controlled study. Neuroreport.

[55] Arendsen L.J., Henshaw J., Brown C.A., Sivan M., Taylor J.R., Trujillo-Barreto N.J., Casson A.J., Jones A.K. (2020). Entraining alpha activity using visual stimulation in patients with chronic musculoskeletal pain: A feasibility study. Front. Neurosci..

[56] Ecsy K., Brown C., Jones A. (2018). Cortical nociceptive processes are reduced by visual alpha-band entrainment in the human brain. Eur. J. Pain.

[57] Köster M., Martens U., Gruber T. (2019). Memory entrainment by visually evoked theta-gamma coupling. NeuroImage.

[58] Mazaheri A., Seminowicz D.A., Furman A.J. (2022). Peak alpha frequency as a candidate biomarker of pain sensitivity: The importance of distinguishing slow from slowing. NeuroImage.

[59] Furman A.J., Thapa T., Summers S.J., Cavaleri R., Fogarty J.S., Steiner G.Z., Schabrun S.M., Seminowicz D.A. (2019). Cerebral peak alpha frequency reflects average pain severity in a human model of sustained, musculoskeletal pain. J. Neurophysiol..

[60] Gulbinaite R., Van Viegen T., Wieling M., Cohen M.X., VanRullen R. (2017). Individual alpha peak frequency predicts 10 Hz flicker effects on selective attention. J. Neurosci..

[61] Ramsay I.S., Lynn P.A., Schermitzler B., Sponheim S.R. (2021). Individual alpha peak frequency is slower in schizophrenia and related to deficits in visual perception and cognition. Sci. Rep..

[62] Yeum T.S., Kang U.G. (2018). Reduction in alpha peak frequency and coherence on quantitative electroencephalography in patients with schizophrenia. J. Korean Med. Sci..

[63] Arjmandi-Rad S., Vestergaard Nieland J.D., Goozee K.G., Vaseghi S. (2023). The effects of different acetylcholinesterase inhibitors on EEG patterns in patients with Alzheimer’s disease: A systematic review. Neurol. Sci..

[64] Vecchio F., Babiloni C., Lizio R., Fallani F.D.V., Blinowska K., Verrienti G., Frisoni G., Rossini P.M. (2013). Resting state cortical EEG rhythms in Alzheimer’s disease: Toward EEG markers for clinical applications: A review. Suppl. Clin. Neurophysiol..

[65] Moretti D.V., Babiloni C., Binetti G., Cassetta E., Dal Forno G., Ferreric F., Ferri R., Lanuzza B., Miniussi C., Nobili F. (2004). Individual analysis of EEG frequency and band power in mild Alzheimer’s disease. Clin. Neurophysiol..

[66] Springer S.D., Wiesman A.I., May P.E., Schantell M., Johnson H.J., Willett M.P., Castelblanco C.A., Eastman J.A., Christopher-Hayes N.J., Wolfson S.L. (2022). Altered visual entrainment in patients with Alzheimer’s disease: Magnetoencephalography evidence. Brain Commun..

[67] https://dancerdesign.co.uk/tactor.html.

[68] Pokorny C., Breitwieser C., Müller-Putz G.R. (2013). A tactile stimulation device for EEG measurements in clinical use. IEEE Trans. Biomed. Circuits Syst..

[69] Giabbiconi C.M., Dancer C., Zopf R., Gruber T., Müller M.M. (2004). Selective spatial attention to left or right hand flutter sensation modulates the steady-state somatosensory evoked potential. Cogn. Brain Res..

